# Twenty-Nail-Dystrophy / Trachyonychia: a case report in a five year old girl seen at the Paediatric Out-patient Department of a Tertiary Hospital in Lafia North-Central Nigeria

**DOI:** 10.11604/pamj.2020.36.380.20936

**Published:** 2020-08-31

**Authors:** Bello Surajudeen Oyeleke, Kure Ibrahim Shekwonyadu

**Affiliations:** 1 Department of Paediatrics, Dalhatu Araf Specialist Hospital Lafia Nasarawa State, Lafia, Nigeria

**Keywords:** Case report, paediatric out-patient, tertiary, Trachyonychia

## Abstract

Twenty-Nail-Dystrophy (TND) also referred to as Trachyonychia is a disorder of the nails. It can affect all the nails hence the name. Trachyonychia is characterized by nail roughness, longitudinal ridging, fragility and hyper-pigmentation. It can occur in all ages and respond poorly to treatment. With this background, we report the case of a five year old girl with this disorder.

## Introduction

Trachyonychiais a nail disoreder that refers to rough nails [1]. It is characterized by longitudinal ridging, fragility and discoloration of all nails [2]. Twenty-Nail-Dystrophy (TND) is another name for Trachyonychia, commonly used when there is involvement of all the twenty nails.[3] It can occur congenital or acquired, the former following mode of inheritance and the latter from other skin diseases [3]. It can run in families in an Autosomal dominant pattern [4]. Diagnosis is made clinically but can be confirmed with biopsy. It can be a chronic disorder that is rarely painful, response to treatment is poor but spontaneous resolution is common [5]. Twenty-Nail-Dystrophy can occur in all age groups but children are commonly affected especially with the idiopathic / familial variety [6]. No sex predilection has been described.

## Patient and observation

**Figure 1:**
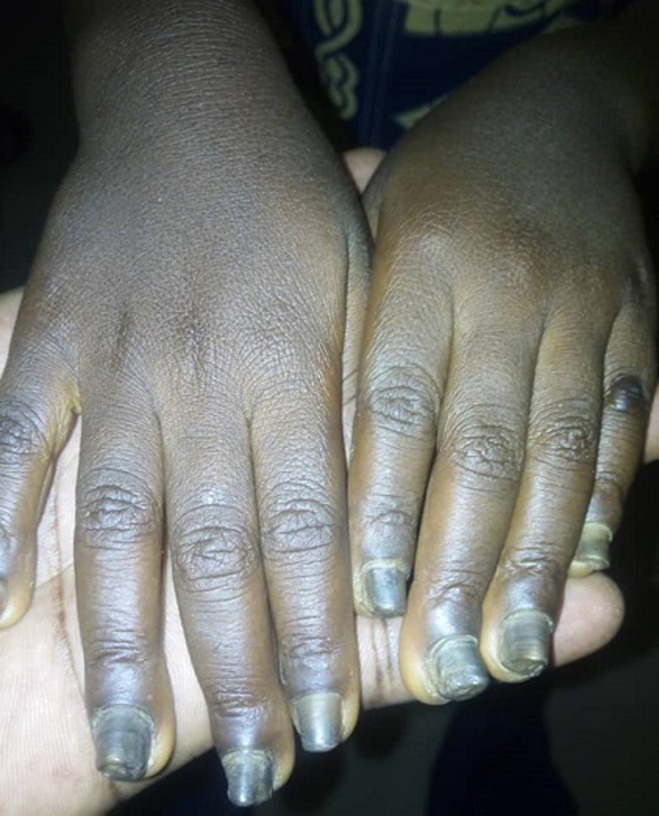
hyperpigmented thickened and rough nails

**Figure 2:**
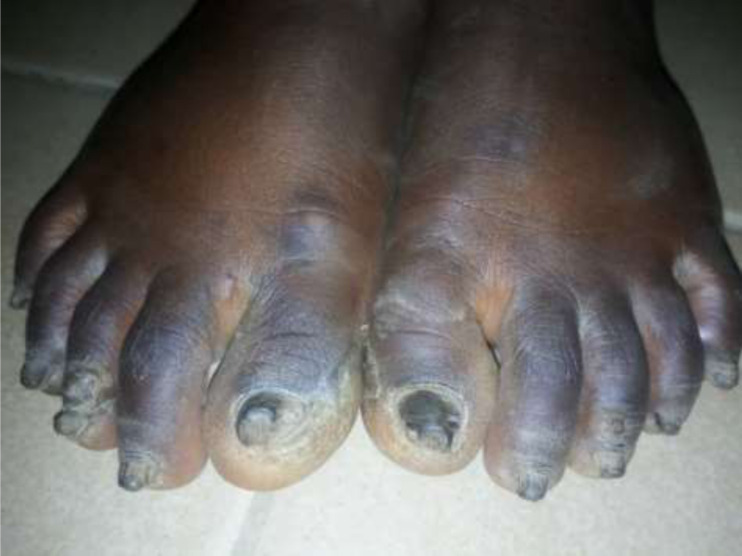
hyperpigmented thickened and rough toe nails

**Figure 3:**
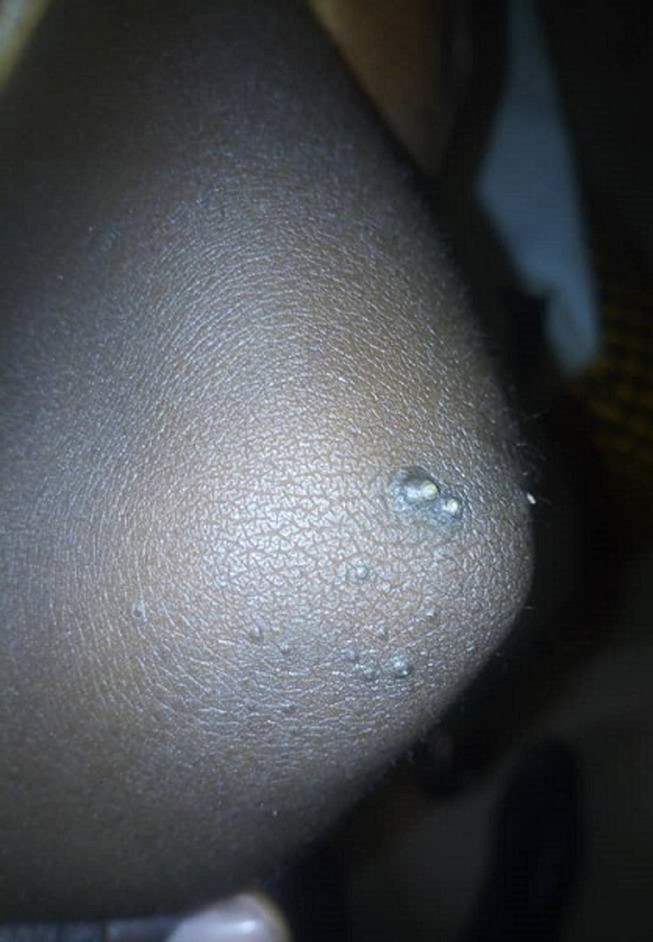
solitary nodule on the elbow

**Figure 4:**
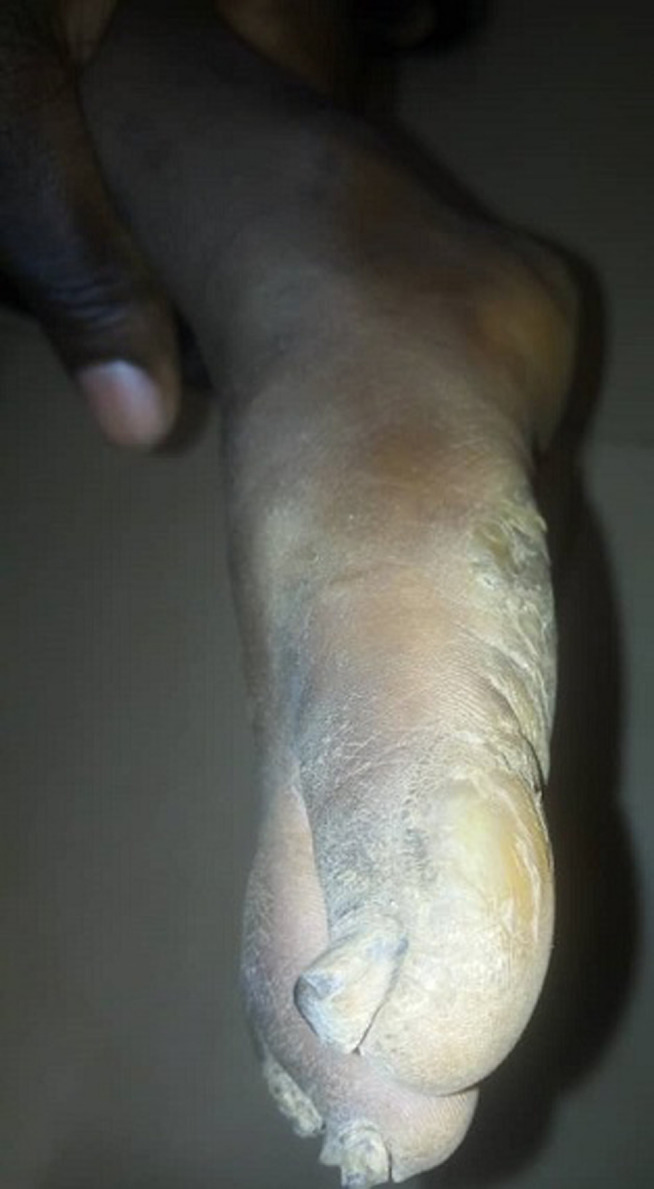
hyperkeratosis on the planter surface

It is with this background that we present the case of a five year old girl who presented to our hospital with history of abnormal nail colour and growth since birth. She was noticed at birth to have dark-brownish to black nail colouration. The nail was then noticed to have become thickened and rough at eight months of age and has worsened progressively involving all the twenty nails. The nails falls-off at about 3-4 months interval, this is more on the finger nails when compared with the toe nails. There is no pain except during the period of nail sloughing. There is associated slight thickness of the skin on the lateral planter surfaces as well as nodules involving the elbows. There are no skin rash, no other skin disorder, no alopecia or tooth involvement. There is no history of trauma and no seasonal variation. There is a similar disorder in the father involving one of his toe nails only. No similar history among her siblings. Parents have presented her to various health centre, medications (both orthodox and traditional) applied without improvement necessitating presentation to us. She´s a product of term gestation and fully immunized according to the National Programme on Immunization (NPI). She´s the fifth and the last of five children in a monogamous family. The father is a junior civil servant while the mother is a petty trader. On examination there is hyper-pigmented thickened rough nails with sand-paper like appearance in all nails (Figure 1, Figure 2). There are 2-3 solitary non tender nodules in both elbows measuring about 1-2 cm each (Figure 3). There is hyperkeratosis of the medial sides of the planter surfaces of the feet (Figure 4). Other systemic examinations are not remarkable. Assessment; Twenty-Nail-Dystrophy. The parent could not afford histology for the above stated reasons. Trial of anti-fungal (topical and systemic), steroids (topical and systemic) as well as antibiotics have not yielded positive result. Attempt at getting others such as Tacrolimus proved abortive. Patient was referred to a center where she can be reviewed and managed by a Paediatric dermatologist but declined and was subsequently loss to follow up.

## Discussion

Our patient is a five year old, which is within the peak age group of 3-12 years reported.[1]Our patient is probably the youngest (5 years) of the patients reported so far, the reported age group from the available literature ranges from ten years to 42 years [1, 3, 5]. This condition can be idiopathic or familial as suggested by the current case, the idiopathic/ familial variant is known to be commoner [6]. Although, there was no reported sex predilection, we found this nail disorder in a female similar to most of the earlier published studies on the subject matter. The TND respond poorly to treatment, this is similar to our finding on this case. There is no family history of diabetes mellitus, similar to earlier report [7]. Balachandrudu et al in India reported a successful response to an immune-modulator Tacrolimus in a 41 year old patient [8].

## Conclusion

Twenty-Nail-Dystrophy is an uncommon disorder of the nails that has an idiopathic cause in most instances. It has a peak age of 3-12 years and responds poorly to treatment. There are more cases among females when compared with the males.

**Recommendation:** high index of suspicion is required in making this diagnosis. Counseling is key in its management due to poor response to therapy.
